# Account of Diseases in the Finsbury Dispensary, from January 20, to February 20, 1805

**Published:** 1805-04-01

**Authors:** J. Reid

**Affiliations:** Grenville-street, Brunswick-square


### *Account of Diseases in the Finsbury Dispensary, from January 20, to February 20, 1805.*

| | | | |
|---|---|---|---|
| Ophthalmia - - - - | 16 | Pneumatosis - - - - | 1 |
| Pneumonia - - - - | 6 | Dyspepsia - - - - - | 12 |
| Catarrhus - - - - - | 10 | Diarrhœa - - - - - | 6 |
| Phthisis - - - - - | 8 | Morbi Cutanei - - - | 10 |
| Asthma - - - - - - | 4 | Morbi Infantiles - - - | 15 |
| Chlorosis - - - - - | 13 | Febricula - - - - - | 7 |
| Amenorrhœa - - - - | 8 | Cephalea - - - - - | 1 |
| Menorrhagia - - - - | 5 | Rheumatismus - - - | 5 |
| Asthenia - - - - - | 17 | Enteritis - - - - - | 1 |
| Anasarca - - - - - | 3 | | |

This

This is the period of the year at which inflammatory complaints are more particularly apt to occur, although at no season do they bear any considerable proportion to that vast mass of maladies with which the present race of mankind are liable to be afflicted.

Diseases have retained their ancient name, whilst they have been altered in their intrinsic character, by a gradual progress of innovation in the fashions, habits, and circumstances, of modern society.

Sydenham was the first physician of his day, and, perhaps, has not been surpassed in reputation or in merit by any previous or subsequent practitioner. But if his mode of treatment were, out of respect to his venerable authority, to be adopted in cases that are now submitted to our professional care, it would prove as deleterious as it formerly was conducive to health and the preservation of existence. It is on this account that that learned lore, on which so many pride themselves, is of little actual service after a man has passed through the elementary stages of a regular medical education. In the productions of classical authors he reads of diseases which he has no opportunity of seeing, and for the cure of which he is never called upon to exercise his science or his skill.

The merely book-taught physician is apt to be misled by the denominations of diseases, with regard to their causes and essential nature. This is, in no instance, better exemplified than in cases of fever. The old-fashioned fevers that were contemporary with Sydenham, and even with Hoffman, were characterized by features of high and active inflammation.

Inflammatory fever, at present, scarcely ever occurs. The febrile affections that now prevail are, in general, marked by an extreme of debility, and every symptom that follows from an exhaustion of the mental and physical energies of the system.

In these latter affections, inordinate and increased action is often conspicuous. The patient not unfrequently exhibits more muscular exertion than would have been accomplished in a condition of health. This, however, is a demonstration not of augmented, but of oppressed power. Extraordinary efforts of this kind are, in general, made during the delirium of typhus, a state in which the depression of strength is particularly remarkable. These fugitive and abrupt exhibitions of morbid energy are very far from indicating the genuine characteristic of strength, which shews itself only in a capacity for regular gular and continued action. The human machinery is of so complicated a structure, and its motions, although various, are all so connected and dependent upon each other, that its derangement in one part may produce a temporarily increased action in the whole machine, in the same manner as a watch chain, if broken, will run down with increased and inordinate force.

Although general affections of an inflammatory nature so very rarely occur, local and organic diseases of this kind are by no means uncommon. A very obstinate and critical case of pneumonia, or inflammation of the lungs, is, at present, under the care of the Reporter; the symptoms of which he has sensibly relieved by repeated bleedings from the arm. Such a state of constitution requires a considerable degree of delicacy in the treatment of it, as debility is an invariable attendant on this disease. But it ought to be considered, that in such cases, bleeding, so far from diminishing strength, is calculated to increase it, by relieving the oppressed vessels from a load of blood, which is disproportionate to the reduced power of propulsion.

This disease, in an early stage, may be counteracted by appropriate remedies; but when, by neglect, or improper management, it is allowed to acquire the exaggerated and alarming physiognomy of phthisis, the physician then has no more to do than to exercise all his talents, merely to sooth anxiety, and to relieve symptoms, without any idea of eradicating the cause of an hopeless and incurable disorder. " Seulement repandre des fleurs sur les bords de notre tombe, et nous masquer l'horreur de ce passage effrayant."*

J. REID.

*Grenville-street, Brunswick-square, March,* 25. 1805.

* Chaptal Annales de Chimie.

MEDICAL

